# HIV-1 Tat Protein Induces the Production of IDO in Human Monocyte Derived-Dendritic Cells through a Direct Mechanism: Effect on T Cells Proliferation

**DOI:** 10.1371/journal.pone.0074551

**Published:** 2013-09-20

**Authors:** Rémi Planès, Elmostafa Bahraoui

**Affiliations:** 1 INSERM, U1043, Toulouse, France; 2 CNRS, U5282, Toulouse, France; 3 Université Paul Sabatier, EA 3038, Toulouse, France; UC Irvine Medical Center, United States of America

## Abstract

During HIV-1 infection, an increase of indoleamine 2,3 dioxygenase (IDO) expression, and dendritic cells (DC) dysfunction were often associated with AIDS disease progression. In this work, we investigated the effect of HIV-1 Tat protein on the expression of IDO, in MoDCs. We show that Tat induces IDO protein expression and activity in a dose dependent manner by acting at the cell membrane. Using Tat-mutants, we show that the N-Terminal domain, Tat 1–45, but not the central region, Tat 30–72, is sufficient to induce the expression of active IDO. Tat protein is also able to induce several cytokines in MoDCs, including IFN-γ, a strong inducer of IDO. In order to understand whether IDO is induced directly by Tat protein or indirectly following IFN-γ production, complementary experiments were performed and showed that: i) at the kinetic level, Tat induced IDO expression before the production of IFN-γ ii) treatment of MoDCs with Tat-conditioned medium was unable to stimulate IDO expression, iii) coculture of MoDCs in a transwell cell system did not allow IDO expression in MoDCs not previously treated by Tat, iv) direct contact between Tat-treated and untreated MoDCs was not sufficient to induce IDO expression in a Tat-independent manner, and v) treatment of MoDCs in the presence of IFN-γ pathway inhibitors, Jak I and Ly294002, inhibited IFN-γ-induced IDO but had no effect on Tat-induced IDO. At the functional level, our data showed that treatment of MoDCs with Tat led to the inhibition of their capacity to stimulate T cell proliferation. This impairement was totally abolished when the stimulation was performed in the presence of 1MT, an inhibitor of IDO activity, arguing for the implication of the kynurenine pathway. By inducing IDO, Tat protein may be considered, as a viral pathogenic factor, in the dysregulation of the DC functions during HIV-1 infection.

## Introduction

Dendritic cells (DCs) play a pivotal role during HIV-1 infection by promoting both dissemination and viral escape. During sexual transmission, HIV-1 particles are captured by DCs, through gp120-DC-SIGN interaction, and transported to the draining lymph nodes, where T4-lymphocytes are infected [1]. DC-HIV-1 interactions are also involved in the immune system dysregulation following modulations of DC phenotypes and functions. A decrease in the ability to activate T cells has been reported [2]. This was linked to a defect in antigen presentation associated with a loss of MHC-II [3] and CD83, CD86 costimulatory molecules [4]. In parallel, DC-HIV-1 interactions are also associated with a great increase of pro-inflammatory cytokines and various immunosuppressive factors including indoleamine 2,3 dioxygenase (IDO) [5]. All these elements contribute to the impairment of an efficient immune response, an impairment that persists during the chronic state.

Identifying the viral factors implicated in DC dysfunction and induced immunosuppressive factors seems to be crucial for understanding the molecular mechanisms of HIV-1 immunopathology and for the development of anti-HIV-1 treatments. One of the potential candidate is HIV-1 Tat protein. Tat is a 14 kDa protein, composed of a single polypeptide of 86 to 101 amino acids, with a transactivating activity. By binding to the TAR (Tat activation region) on the nascent viral RNA, Tat protein recruits various cellular factors, including cyclin T1 and CDK9, to form TAK (Tat associated complex kinase) which is essential for the elongation of viral transcripts [6]. At structural level, Tat contains six identifiable domains, including the cystein-rich (aa 20–31), the core (32–47) and the basic (49–57) domains, which are essential for the transactivating activity [7]. The basic domain of Tat is also essential for Tat internalization and nuclear localization [8]. Despite the absence of signal peptide, Tat protein is secreted, as an early gene product, by infected cells. The protein released can then be taken up both by infected cells to transactivate HIV-1 replication and by uninfected cells to modulate various functions [9]. Secreted HIV-1 Tat has been found as soluble protein in the sera of HIV-1-infected patients at nM levels (0.1 to 4 nM) [10], [11]. However, these concentrations are probably underestimated, and are most likely higher in the neighbouring infected cells.

At functional level, several reports have shown that Tat protein has numerous effects, including production of pro- and anti-inflammatory cytokines TNF-α [12], IL-6 [13], [14], IL-1β [15], IL-12 [16], IL-10 [17]–[19], chemokine receptor increase CXCR4 [20] and CCR5 [21] and apoptosis of T-lymphocytes [22], [23]. Thus, by affecting the production of these factors and others (review in [9], [24], [25]), HIV-1 Tat protein might play a key role in viral pathogenesis. In this study, we focused on one potential immunosuppressive mechanism involving catabolism of tryptophan, an essential amino acid, by IDO following its induction by HIV-1 Tat protein in dendritic cells [26].

Human IDO is an intracellular monomeric protein of 45 kDa, with oxygenase activity that catalyzes the cleavage of L-tryptophan into N-formyl-kynurenine. Subsequently, kynurenine is catabolyzed by a range of other enzymes constitutively expressed to lead to the production of other important metabolites such as hydroxykynurenine, quinolinic acid, and Kynurenic acid [27], [28]. However, IDO is still the rate limiting enzyme in the kynurenine pathway. While IDO is present in several cells, mainly in macrophage and dendritic cells, another oxygenase, named TDO (tryptophan 2,3-dioxygenase), with a more strict specificity for tryptophan degradation, is present essentially in the liver [29]. TDO is a homotetrameric protein of 134 kDa that regulates plasma levels of tryptophan. Its expression is induced by tryptophan, tyrosine, histidine and kynurenine. IDO is a more regulated gene product that is inducible in antigen presenting cells, essentially by two major cytokines, IFN-γ and TGF-β, acting through Jak/stat and PI3K respectively [30]–[32].

Early reports underlined the antimicrobial action of IDO, essentially on microorganisms such as *Clamydia pneumoniae* [33] and *toxoplasma gondii* [34], which are unable to synthesize their own tryptophan. This observation can be related, at least in part, to the capacity of IFN-γ, a strong inducer of IDO, to block the growth of the parasite [34]. In addition to its involvement in defense against pathogens, an accumulation of recent data highlights the immunoregulatory properties of IDO on T-cell proliferation [35]–[37], apoptosis [5], [35] and Treg differentiation [31], [38]–[40].

During HIV-1 infection, an increase of tryptophan catabolism has been shown in the plasma of HIV-1 infected patients [41]. This increase was observed more in HIV-1 non-controllers (CD4-T cells less than 350/µl and viral load >10 000 RNA copies/ml) than in HIV-1 controllers (CD4-T cells more than 500/µl and viral load <2000 RNA copies/ml) [5]. The increase in tryptophan catabolism was associated with a substantial expression of IDO in lymph nodes and gastro intestinal mucosa [5]. The expression of IDO was found predominantly in myeloid dendritic cells [5]. Other studies have also reported increased expression of IDO in macrophages [42] and plasmacytoid dendritic cells [43]. This IDO activity is associated with immunological disorders including, inefficient immune response, T cell exhaustion and neurological dysfunction and injury [44], [45]. As shown for TNF-α [46], [47], a proinflammatory cytokine, and IL-10 [48]–[50], a highly immunosuppressive cytokine, IDO expression/activity also seems to parallel AIDS disease progression. Thus, an important issue is to identify the direct and/or indirect viral factors involved in the expression and activation of the IDO pathway. Different viral proteins have been reported to be involved, including the envelope glycoprotein gp120 [43], and the regulatory proteins Nef and Tat [51]–[53], which could act directly or indirectly via the induction of proinflammatory cytokines. In macrophages, IDO expression by HIV-1 infection is known to be mediated by IFN-γ production [42]. In plasmacytoid dendritic cells (pDC), in vitro exposure to HIV-1 stimulates IDO expression after direct attachment of gp120 to CD4, or alternatively by inducing TLR7 pathway and IFN-γ production [43], [54]. Although some studies have reported the implication of HIV-1 Tat protein in the induction of IDO [52], the mechanism of this induction and its effect on T-cell proliferation have not been investigated. These two important questions constitute the aim of the present study.

## Materials and Methods

### Ethics Statement

This study was approved by the Research Ethical Comity Haute-Garonne.

Human Peripheral blood mononuclear cells were isolated from buffy coat, from healthy donors. Buffy coats were provided anonymously by the EFS (établissement français du sang, Toulouse, France). Written informed consents were obtained from the donors under EFS contract N° 21/PVNT/TOU/INSERM01/2011-0059, according, to “Decret N° 2007-1220 (articles L1243-4, R1243-61)”.

### Materials

#### Tat protein

Recombinant HIV-1 Tat protein (aa 1–86) from HIV-1 Lai strain was obtained from “Agence Nationale de la Recherche sur le SIDA” (Paris, France), glutathione S-transferase (GST), GST-Tat full length protein (1–101) from HIV-1 strain SF2 or deleted mutants GST-Tat 1–45 and GST-Tat 30–72 were produced and purified in our laboratory as previously described [17]. The level of endotoxin in all these recombinant proteins was assessed using the Limulus amoebocyte lysate assay (Bio-Sepra, Villeneuve la Garenne, France) and was shown to contain less than 0.3 EU/µg, the limit of detection of this test.

#### Chemical products

LPS, from E. coli serotype R515, was purchased from Alexis biochemicals. Chemical inhibitors of phosphoinositide 3-kinases (LY 294002) and Janus kinase inhibitor (JAK Inhibitor I) were purchased from Calbiochem. L-tryptophan, L-Kynurenine, 1-Methyl Tryptophan (1MT), DMSO and Ehrlich’s reagent were from Sigma-Aldrich. CellTrace CFSE Proliferation kit was purchased from Invitrogen.

#### Recombinant cytokines and antibodies

Recombinant human IFN-γ and TNF-α cytokines were purchased from eBioscience. Recombinant GM-CSF and IL-4 were from HumanZyme. Anti-human-IDO, Mab, were purchased from Abcam. Secondary rabbit antibodies coupled with HRP were from Dako and those coupled with APC, produced in goat, were purchased from Abcam. Anti-β-actine, AC-15, Mab, were purchased from Sigma-Aldrich. Anti-CD3, OKT3, Mab, and anti-CD11c-FITC were from eBioscience. Anti-IL-10, #25209, Mab, were purchased from R&D system. Anti-mouse IgG2a Alexa Fluor 633 were from Invitrogen. Fluorochrome-conjugated antibodies anti-CD1a-FITC, anti-CD14-PE, anti-CD80-FITC, anti-CD86-PE, anti-CD83-FITC, anti-HLA-DR-FITC and isotype control were from Biolegend. Anti-Tat antibodies were obtained from ANRS. Anti-GST antibodies were produced in our laboratory as described by [55].

### Generation of Monocyte-derived Dendritic Cells

Peripheral blood mononuclear cells (PBMCs) were isolated from buffy coats of healthy blood donors (EFS, Toulouse) by centrifugation on Ficoll paque (GE Healthcare). Monocytes were isolated by adherence to tissue culture plastic on 6-well plates (Beckton Dickinson) for 1 h at 37°C in 5% CO_2_. Non-adherent cells were removed and adherent cells were washed three times with PBS, then used for the generation of dendritic cells. When analyzed by flow cytometry, more than 94% of this adherent population was CD14^+^. To allow them to differentiate into monocyte-derived dendritic cells (MoDCs), CD14^+^ cells were cultured in RPMI medium (Invitrogen) supplemented with 10% FCS (Invitrogen), containing penicillin (100 IU/ml) and streptomycin (100 µg/ml), 10 ng/ml recombinant granulocyte macrophage-colony-stimulating factor (GM-CSF) and 10 ng/ml interleukin-4 (IL-4). Alternatively, monocytes were also isolated by positive selection using a CD14+ isolation kit (Miltenyi biotec). After 5 days of culture, loosely adherent cells were recovered by gentle pipetting and used as immature dendritic cells in our experiments. Over 90% of cells had the standard phenotype of immature dendritic cells: CD1a+, CD14−, CD80+, CD86+, CD83−, HLA-DR+.

### Treatment of Monocyte-derived Dendritic Cells with Tat

At least 1 hr before treatment, MoDCs were resuspendend in RPMI complete medium (supplemented with 10% FCS containing penicillin (100 IU/ml) and streptomycin (100 µg/ml) at 1.10^6^ cells/ml in 6-well plates. Cells were then treated with Tat protein or its derivatives, in the presence or absence of inhibitors for a period of 24 h or alternatively as indicated. Cell culture supernatants were collected and kept frozen until cytokine quantification, while cells were recovered and used for the quantification of IDO expression and activity. For signalling pathways blockade, MoDCs were treated with chemical inhibitors for 30 min before stimulation with Tat or IFN-γ.

To obtain Tat conditioned medium, MoDCs were treated with Tat for 1 h and then washed three times with PBS to eliminate soluble Tat. Culture was then conducted for 24 h. Cell supernatant was recovered, centrifuged for 10 min at 1200 rpm and the supernatant was used directly as Tat conditioned medium.

The transwell experiments were performed in 6-well plates (Becton Dickinson). Untreated MoDCs were cultured in the lower compartment. In the upper chamber of a 1-µm transwell insert (from Becton Dickinson) we added autologous MoDCs previously incubated with Tat for 1 h and washed three times with PBS. Cells were kept in co-culture in the transwell for a further 24 h.

In a direct co-culture experiment, we mixed CFSE-labelled MoDCs with autologous unlabelled MoDCs previously treated with Tat for 1 h. After three washes and 24 h of incubation, MoDCs were recovered and CFSE-labelled and unlabelled MoDCs were separated by cell sorting using FACSAria II (Becton Dickinson) and analyzed separately for IDO expression.

### Analysis of IDO Expression and Activity

IDO protein expression in MoDCs was investigated by immunoblot analysis. MoDCs previously stimulated or not by different ligands, were lyzed by 20-min treatment in lysis buffer (20 mM Tris-HCl, 150 mM NaCl, 1 mM EDTA, 0.5% NP-40, 0.2% SDS, pH 7.4 and containing a protease inhibitor cocktail) on cold. Protein concentrations in cellular extracts were determined by Bradford assay. For the analysis of IDO expression, equal amounts of protein (80–100 µg) were separated by 12% SDS-PAGE and then transferred to nitrocellulose membrane. Membranes were saturated in Tris-buffered saline (TBS) with 0.05% Tween 20 containing 5% non-fat milk (pH8) for 1 hr and then incubated, overnight, with anti-human IDO antibodies (3 µg/ml) at 4°C. After three washes with TBS 0.1% Tween 20, the membranes were further incubated with a secondary antibody (rabbit anti-mouse IgG HRP) for 1 h at room temperature. After three washes, immunoreactive bands were detected with a chemiluminescent substrate (Pierce). To control the protein load, membranes were first dehybridized by incubation in glycine 0.1 M, 0.1% NP40, 1% SDS, pH 2.2 buffer for 20 minutes and then used for β-actin detection by using the anti-β-actin, AC-15, Mab.

For intracellular detection of IDO by flow cytometry on a FACSCalibur (Becton Dickinson), MoDCs were first washed once with PBS, 5 mM EDTA, and then once with PBS, 5% FCS. Cells were then incubated for 30 min on cold with anti CD11c-FITC (1/10). After 2 washes with PBS 5% FCS, cells were treated for intracellular IDO labelling using the intracellular staining kit from BD Bioscience according to the manufacturer’s instructions. For IDO detection, intracellular staining was performed by an indirect labelling assay using a primary mouse anti-human IDO (1/100) for the first step and a goat anti-mouse IgG2b-APC conjugated antibody (1/200) for the second step.

To evaluate the activity of IDO in catabolizing tryptophan into kynurenine, MoDCs were resuspended in Hanks buffered saline solution (HBSS) supplemented with 500 µM L-tryptophan and incubated for 2 to 4 hr at 37°C. Supernatants were harvested and kynurenine was quantified by Ehrlich’s Assay. Briefly, supernatant was cleared of its protein contents by treatment with 30% trichloroacetic acid followed by 5 min of centrifugation at 10000 rpm. Then, 100 µl of soluble phase was mixed with 100 µl of Ehrlich’s reagent (25 mg/ml of 4-dimethylaminobenzaldehyde in glacial acetic acid) in 96-well plates. The OD was measured at 492 nm and kynurenine concentrations were calculated using a kynurenine standard curve.

### T-cell Proliferation Assay

In the T-cell proliferation assay, the non-adherent fraction of PBMC, or CD14 negative untouched cells, were used as Peripheral Blood Lymphocytes (PBL). These cells were first labelled with 2 µM CellTrace CFSE Proliferation kit (Invitrogen). Labelled PBL were then cocultured with autologous MoDCs in round-bottomed 96-well plates at the ratio of 4.10^5^ PBL for 2.10^5^ MoDC. Before PBL was added to the coculture, MoDCs were either treated with the IDO inhibitor 1MT for 2 hr or left untreated. T-cell proliferation was stimulated by anti-CD3, OKT3, Mab (10 ng/ml) in a total volume of 200 µl of RPMI complete medium. At day 5 post activation, cells were harvested and labelled with anti-human CD3 used as primary antibody followed by detection with anti-mouse IgG2a Alexa Fluor 647 conjugated secondary antibody and analyzed by flow cytometry.

### Antibodies, Cytokines and Cytokine Quantifications

Cytokine quantification of TNF-α, IL-10, IL-6, IFN-α1, IL-12p70 and IFN-γ in the cell’s supernatants was performed using a specific ELISA kit from eBioscience. Briefly, the first monoclonal antibody was used for capture overnight at 4°C. After three washes with PBS containing 0.05% Tween 20 (wash buffer), plates were saturated by adding 250 µl of a protein solution (diluent assay) for one hr at room temperature. After three washes, culture supernatants (100 µl/well) were added and incubated for 2 hr at room temperature. Plates were then washed three times and incubated for 1 hr at room temperature with a biotinylated anti-cytokine antibody. After five washes, the bound biotinylated antibody was detected by an additional 30 min incubation with streptavidin peroxidase. After seven washes, plates were incubated with the enzyme substrate (TMB). The reaction was stopped by adding 50 µl of H2SO4 (4N) to each well. Absorbance was read at 450 nm with a wavelength correction at 570 nm. Cytokines were quantified from a standard curve generated by using various concentrations of recombinant protein of each cytokine. The limit of detection of each cytokine was 4 pg/ml for TNF-α, 2 pg/ml for IL-10, 2 pg/ml for IL-6, 15 pg/ml for IFN-α1, 4 pg/ml for IFN-γ and 4 pg/ml for IL-12p70.

### Statistical Analysis

The Mann-Whitney non parametric test was used in this study.

## Results

### Tat Protein Induces the Production of IDO in Human Monocyte Derived Dendritic Cells

**Figure 1:**
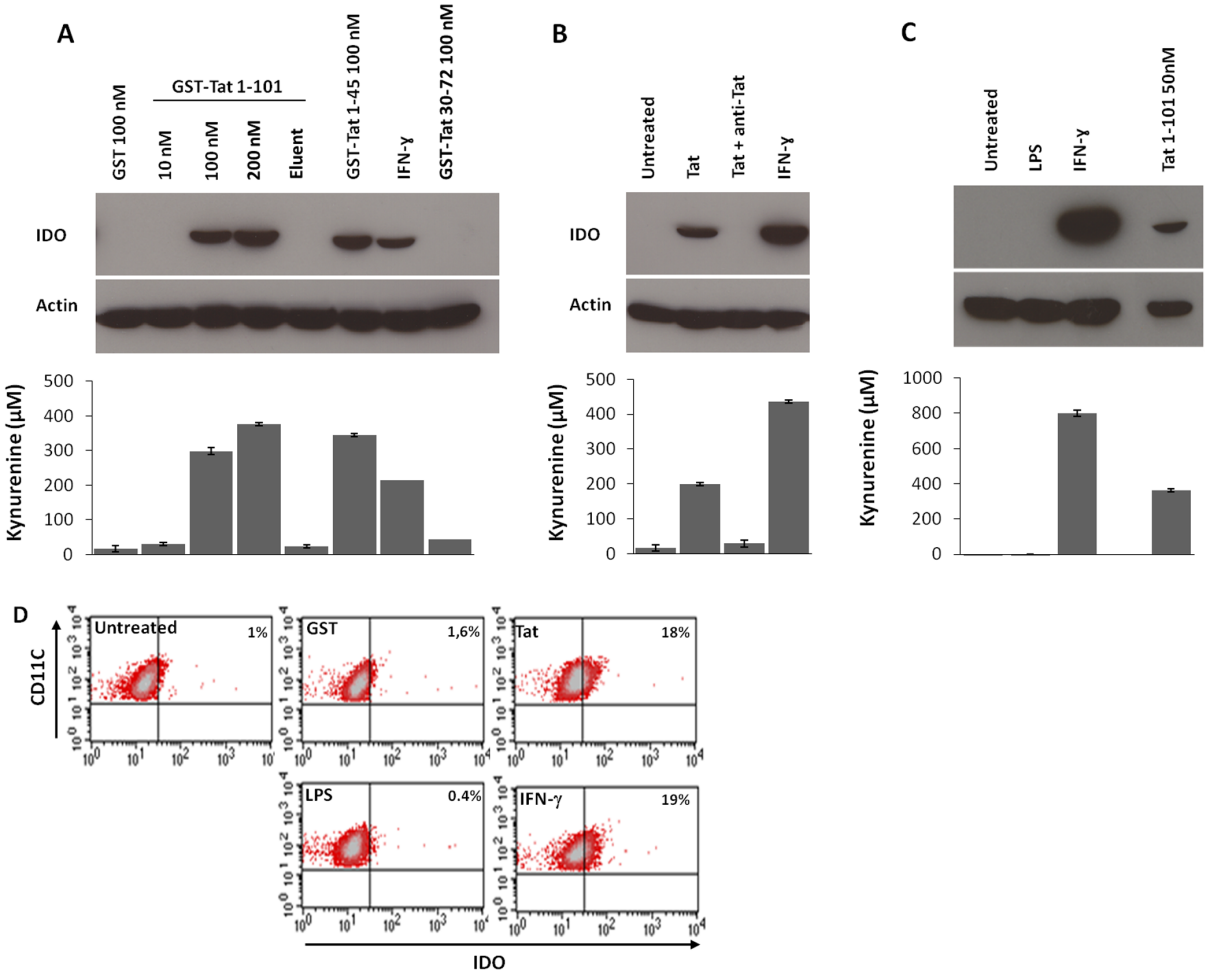
HIV-1 Tat induces IDO protein expression and activity in MoDCs. Tat protein specifically induces IDO expression/activity in MoDCs. (A) MoDCs (2.10^6^) were treated with 50 nM of Tat 1–86 protein (Lai strain) for 24 hr. Untreated and IFN-γ-treated (500 ng/ml) cells were used as negative and positive controls respectively. The specificity of Tat was evaluated by treating MODCs with Tat (50 nM) previously incubated with anti-Tat antibodies (3 µg/ml) for 30 min at 37°C. (B) MoDCs (2.10^6^) were treated with increasing amounts (10, 100, and 200 nM) of full length recombinant GST-Tat 1–101 (SFII strain) or with the truncated forms GST-Tat 1–45, and GST-Tat 30–72 (100 nM). GST protein alone (100 nM) and IFN-γ (100 ng/ml) were used as negative and positive controls respectively. Eluent corresponds to the fraction of GST-Tat not retained following incubation of GST-Tat (100 nM) with anti-Tat/anti-GST coupled to protein A sepharose beads (pharmacia biotech). (C) MoDCs (2.10^6^) were treated with LPS (1 µg/ml), IFN-γ (1 µg/ml), GST-Tat 1–101 (50 nM), or kept untreated for 24 hr. For each experiment, the upper panel shows IDO protein expression by immuno-blot, and the loading control (β-actine) in the second line. Lower panels’ shows the tryptophan catabolism activity determined by Ehrlich’s spetrophotometric assay. (D) Intracellular IDO protein expression was assessed by flow cytometry in CD11c positive MoDCs after stimulation for 24 hr with GST (100 nM), GST-Tat (100 nM), LPS (1 µg/ml) IFN-γ (500 ng/ml), or untreated MoDC. The settings were made on the control isotype. Data are representative of three to four independent experiments.

To investigate the role of Tat in the induction of IDO, immature MoDCs (Figure S1) were treated with HIV-1 Tat 1–86 from the Lai strain or with GST-Tat 1–101 from the SF2 strain. Both Tat proteins induce the expression of IDO in a dose dependent manner (Figure 1A). IDO expression was specific to Tat as shown by its inhibition when the stimulation was performed in the presence of anti-Tat antibodies (Figure 1B). In agreement with the implication of Tat protein, fraction that was depleted of GST-Tat protein (Eluate) with anti-Tat/GST antibodies (Figure S2 A) became unable to induce IDO (Figure 1A). As a positive control, we showed that treatment of MoDC by IFN-γ, a potent IDO inducer, stimulated a clear expression of IDO, while no evident IDO detection was observed with LPS treatment (Figure 1C). As a negative control, no IDO expression was obtained when MoDC cells were stimulated in the same conditions with GST alone (Figure 1A). It is interesting to note that no detectable IDO production was observed in non-stimulated cells. This result indicates that IDO expression is observed only after its stimulation by specific inducers such as IFN-γ or HIV-1 Tat.

We next analysed the intracellular induction of IDO by Tat using flow cytometry. In agreement with SDS-PAGE and WB data, we showed that, like IFN-γ (19% IDO positive cells), Tat protein stimulated IDO expression (18% IDO positive cells), while the percentage of IDO positive cells stimulated by GST (1.6% IDO positive cells) or LPS (0.4% IDO positive cells) remained comparable to that of untreated cells (1% IDO positive cells) (Figure 1D).

To assess whether the Tat induced IDO was enzymatically active, we measured its activity to oxidize L-Tryptophan to kynurenine using a colorimetric assay. The results presented in Figure 1 (A to C) show that the induction of IDO expression, as indicated in Western blot data (upper panels), was associated with kynurenine increase (lower panels) while no enzymatic activity was observed in the culture medium of untreated, LPS- or GST-treated cells. As expected, treatment of cells with IFN-γ led to a significant increase in kynurenine (Figure 1 A–C). Altogether, our data show that HIV-1 Tat protein induces a biologically active IDO in a specific manner.

### HIV-1 Tat Protein Induces IDO by Acting at the Cell Membrane Level

Tat protein contains a basic domain between amino acids 49 and 57 which is responsible for Tat internalization and its nuclear localization. Thus, Tat protein could induce IDO expression by acting either at the cell membrane or intracellularly. To investigate the mechanism involved, the N-terminal fragment GST-Tat1-45 and the central fragment GST-Tat 30–72 were used, in addition to the total GST-Tat1-101 protein, for MoDC stimulation. Similar to Tat protein, GST-Tat 1–45, but not GST-Tat 30–72 or GST alone, activated the expression of IDO (Figure 1A). In contrast to IDO induction, only the full length GST-Tat 1–101 shows optimal HIV-1 LTR transactivation activity in HeLa cells stably transfected with the gene of β-galactosidase on the control of the HIV-1 LTR promoter (Figure S2 B). These results show that, despite the absence of the basic region 49–57, which is essential for the penetration of Tat, the N-terminal fragment Tat 1–45 is sufficient to stimulate the expression of IDO. This clearly demonstrates that Tat protein mediates IDO induction by acting at cell membrane level.

### Mechanisms of Tat Induced IDO: Direct or Indirect

**Figure 2:**
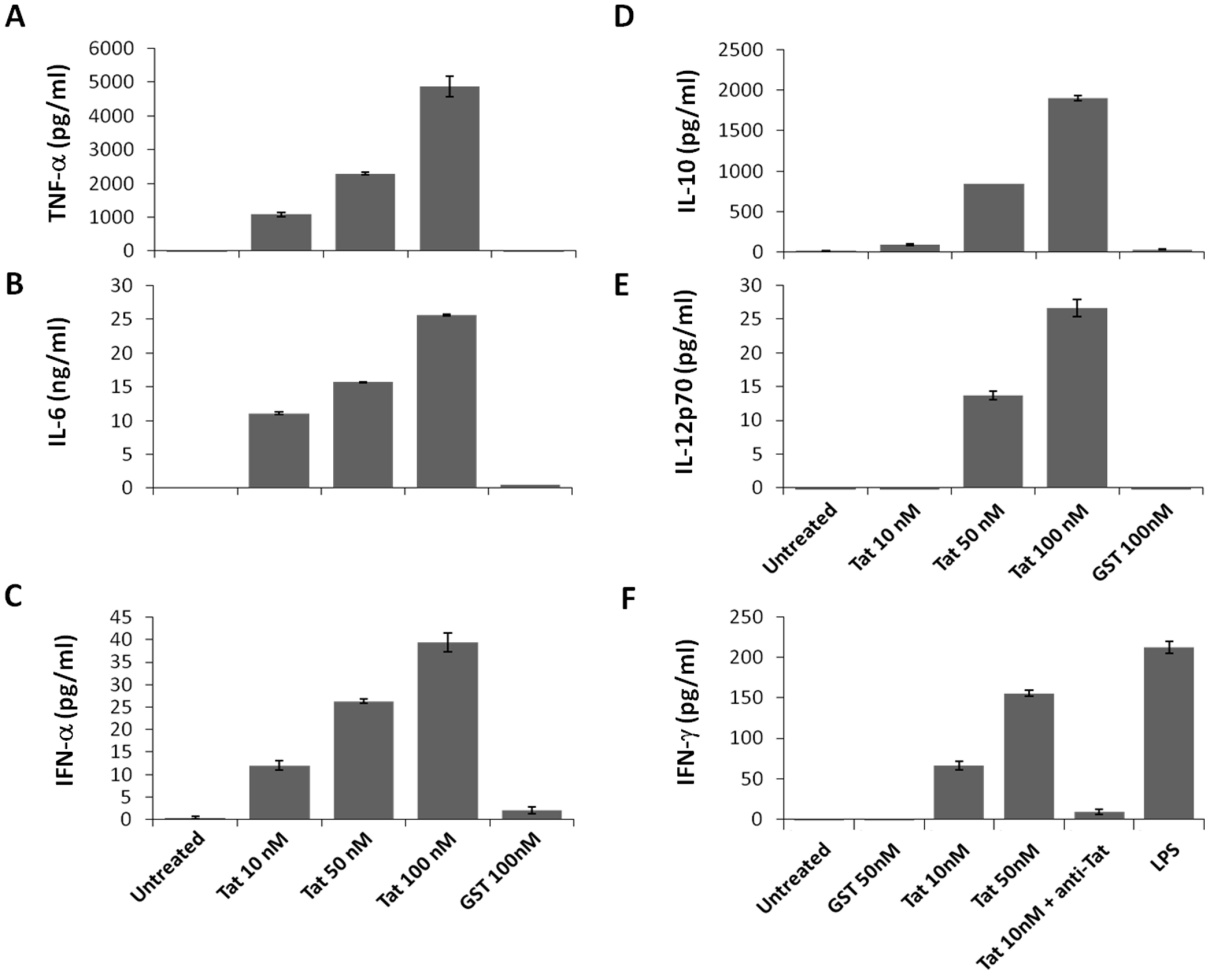
Tat induces the production of TNF-α, IL-6, IL-10, IL-12, IFN-α1 and IFN-γ in MoDCs. MoDCs (0.5×10^6^) were incubated with increasing amounts of GST-Tat 1–101 protein (10, 50 and 100 nM). Untreated and GST-treated cells were used as controls. The capacity of Tat to induce IFN-γ was tested in the presence of anti-Tat antibodies. Untreated and LPS-treated MoDC were used as negative and positive controls respectively. After 24 h, cell supernatants were harvested and analyzed for cytokine production by ELISA, including (A) TNF-α, (B) IL-10, (C) IL-6, (D) IL-12p70, (E) IFN-α1 and (F) IFN-γ.

**Figure 3:**
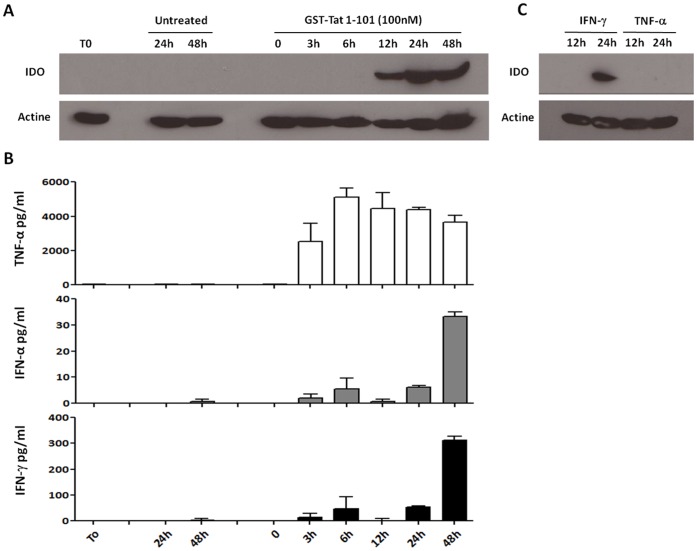
Kinetics of IDO induction and cytokine production by Tat in MoDCs. (A) MoDCs (2.10^6^) were incubated with full length GST-Tat protein 100 nM, IFN-γ (100 ng/ml), TNF-α (10 ng/ml) or kept untreated for the indicated time. Afterwards, IDO protein expression was assessed by Western blot in cell extracts. (B) The production of cytokine (TNF-α, IFN-α and IFN-γ) at different time points was measured in cell supernatants by ELISA.

Tat protein can exert its action to stimulate the production of IDO by acting directly or indirectly *via* the production of cytokines. With these possibilities in mind, we first explored the panel of Tat-induced cytokines known for their potential to induce IDO. We showed that Tat protein was able to stimulate the production of TNF-α, IL-10, IL-12, IL-6, IFN-α and IFN-γ (Figure 2A–F). The production of these cytokines is specific to Tat as shown by the absence of cytokine production when MoDCs were stimulated with GST alone (Figure 2A–F). Among these cytokines, only IFN-γ is known to be able to stimulate the production of IDO. For this reason, we further characterized the specificity of Tat to induce IFN-γ by showing that, when the stimulation of MoDCs was performed in the presence of anti-Tat antibodies IFN-γ, production was totally inhibited (Figure 2F). Thus we showed, as expected, that IFN-γ, but not TNF-α, is capable of stimulating the production of IDO (Figure 3A).

**Figure 4:**
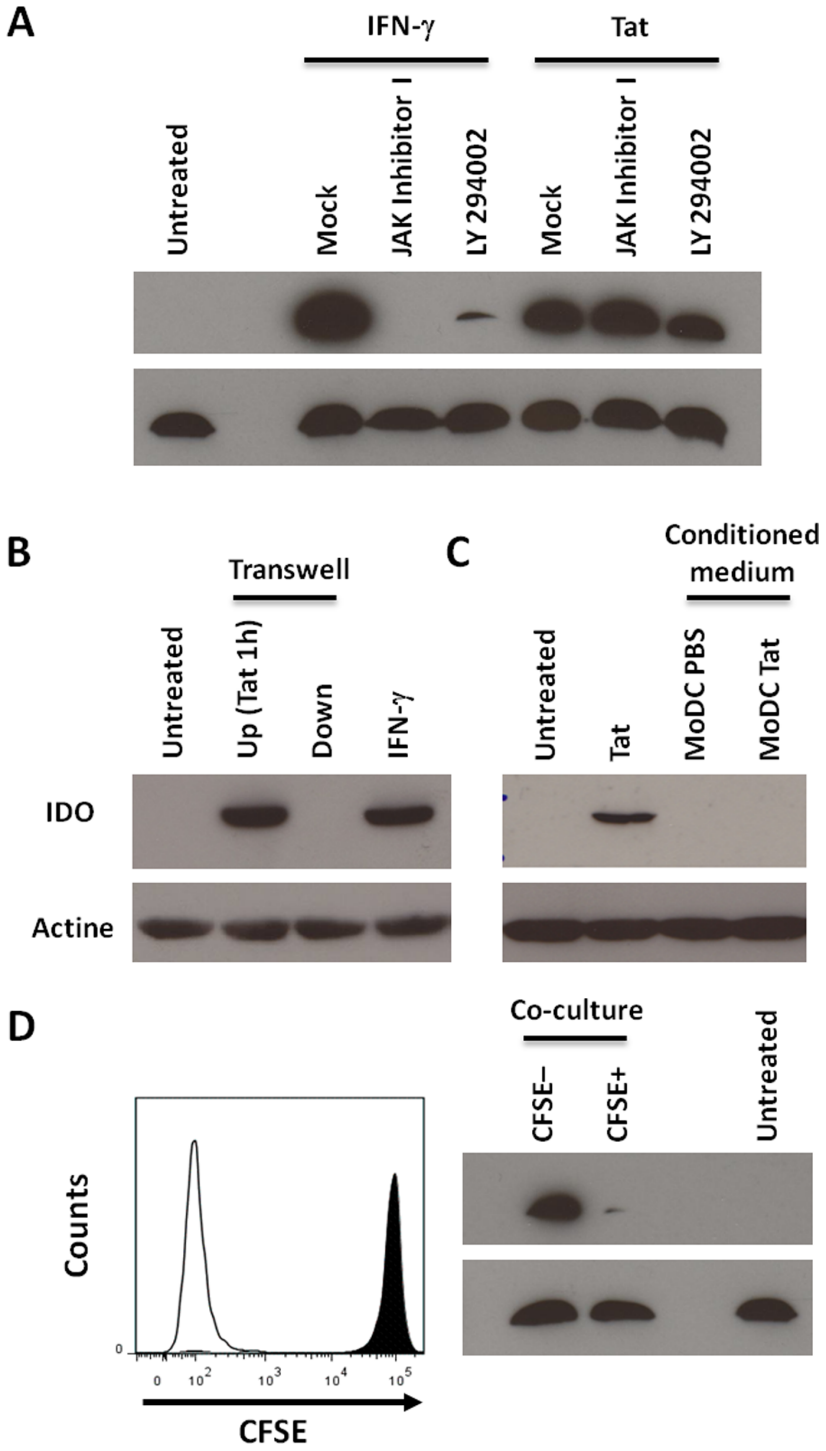
Tat stimulates IDO expression via a direct mechanism. In order to decipher the mechanism used by Tat to induce IDO protein expression, four different protocols were used. In (A) the role of Janus kinase and PI3K signalling pathways in Tat-induced IDO protein expression was evaluated: MoDCs were treated with Tat protein (100 nM) or IFN-γ 500 ng/ml) in the presence or absence of 1 µM Janus kinase inhibitor (JAK Inhibitor I) or 20 µM phosphoinositide 3-kinases (LY 294002) inhibitor, IDO protein was detected in MoDC extract by Western blotting experiments, and β-actine was used as loading control. Data are representative of three independent experiments. In (B) autologous Tat-treated (2.10^6^) or untreated MoDCs (2.10^6^) separated by a 1 µM transwell insert were put in co-culture for 24 h. In the bottom part, MoDCs were untreated and, in the upper chamber, MoDCs had been previously treated with Tat 100 nM for 1 h. MoDCs alone and direct IFN-γ (100 ng/ml) stimulation were used as negative and positive controls respectively. In (C) MoDCs (2.10^6^) were incubated in medium conditioned by Tat 100 nM or PBS for 24 hr. Unconditioned medium or direct treatment by Tat (50 nM) were used as controls. In (D) autologous MoDCs (10^6^) treated, or not, by Tat for 1 h and washed were kept in direct coculture. To discriminate between the two conditions, untreated cells were previously labelled with CFSE (1 µM) whereas Tat-treated cells were kept unlabelled. After 24 h, CFSE positive and CFSE negative MoDC were sorted (lower panel) and analyzed separately for IDO expression.

One can wonder whether the IDO production was mediated directly by Tat action or indirectly *via* Tat-induced IFN-γ. To explore the mechanism involved, complementary approaches were used. We compared the kinetics of IDO production induced by Tat and IFN-γ. The results presented in Figure 3 show that IDO became detectable after 12 h of stimulation by Tat (Figure 3A), while the induction of IDO by IFN-γ is induced only after 24 hr of stimulation (Figure 3A). In contrast, TNF-α has no effect on IDO induction even after 24 h of stimulation (Figure 3A). We next analysed the kinetic of cytokine secretion. Tat-induced IFN-γ and IFN-α are significantly produced only after 24 h of Tat treatment (Figure 3B), while TNF-α which is shown to be unable to stimulate IDO production (Figure 3A) is detectable as early as 3 hr post-Tat stimulation and reach the maximum after 6 h of treatment (Figure 3B). In agreement with a direct implication of Tat protein in IDO induction, we showed that, when MoDCs were stimulated in the presence of the inhibitors of the IFN-γ pathway: Jak I, an inhibitor of Janus tyrosine kinase Jak, and Ly 294002, an inhibitor of PI3K, production of IDO was totally or strongly inhibited when the stimulation of MoDCs was performed with IFN-γ (Figure 4A), while these inhibitors had no effect on the capacity of Tat to induce IDO. As controls, treatment of MoDCs with Jak I and Ly 294002 chemical inhibitors or DMSO solvent had no effect on IDO expression and cell cytotoxicity (Figure S3 A–B). Although these data argue for a direct implication of Tat, we cannot exclude the possibilities that, on the one hand, a very low dose of IFN-γ, undetectable by our assay, remained sufficient to induce IDO or, on the other hand, another cytokine, not explored in our panel of Figure 2 A–F, was involved.

To determine whether these possible explanations could be excluded, the expression of Tat- induced IDO by MoDCs was analyzed by culturing cells in a transwell co-culture system that allowed factor diffusion between the upper and lower chambers. MoDCs previously treated by Tat and washes were cultured in the upper chamber and untreated MoDCs were cultured in the lower chamber. After 24 hr of coculture, MoDCs from each compartment were harvested and tested by Western blot for IDO expression. Figure 4B shows, as expected, a clear induction of IDO expression in MoDCs that had been previously treated by Tat, while no IDO induction is observed in cells from the lower chamber that were in contact with the medium only (Figure 4B). Taken together, these results suggest that the production of IDO by Tat requires a direct contact of Tat protein with human dendritic cells. This conclusion is also in agreement with the incapacity of Tat-conditioned medium to induce IDO in MoDCs (Figure 4C). However, this does not exclude the possibility that treatment of MoDCs by Tat stimulates new cell membrane factors which in turn can also stimulate the production of IDO in non-Tat-treated cells, following cell-cell interactions.

To investigate this third hypothesis we set up the following protocol. MoDCs were treated with Tat for 1 hr and cocultured with an equivalent fraction of Tat-untreated cells that were labelled with CFSE. 24 hr later, CFSE labelled and unlabelled MoDCs were separated by cell sorting (Figure 4D left panel) and IDO production was analyzed by Western blot in each fraction. The results depicted in Figure 4D (right panel) show as expected, the presence of IDO in unlabelled cells while, despite cell-cell contact, no induction of the enzyme expression is observed in CFSE labelled MoDC (Figure 4D). These data demonstrate that cell-cell interaction is not sufficient to stimulate IDO induction in non-Tat-treated cells and suggest that induction of IDO in MoDCs is rather mediated by a direct action of HIV-1 Tat protein following its action at cell membrane level.

### Effect of Tat-induced IDO on the Capacity of Dendritic Cells to Stimulate T cell Proliferation

The results presented above show that Tat protein, by acting at the cell membrane level, induced the production of IDO. This enzyme is known for its capacity to oxidize tryptophan to different metabolites including kynurenine, 3-hydroxykynurenine, and 3-hydroxyanthranilic acid. These metabolites are involved in various functions, including inhibition of cell proliferation, differentiation and apoptosis.

**Figure 5:**
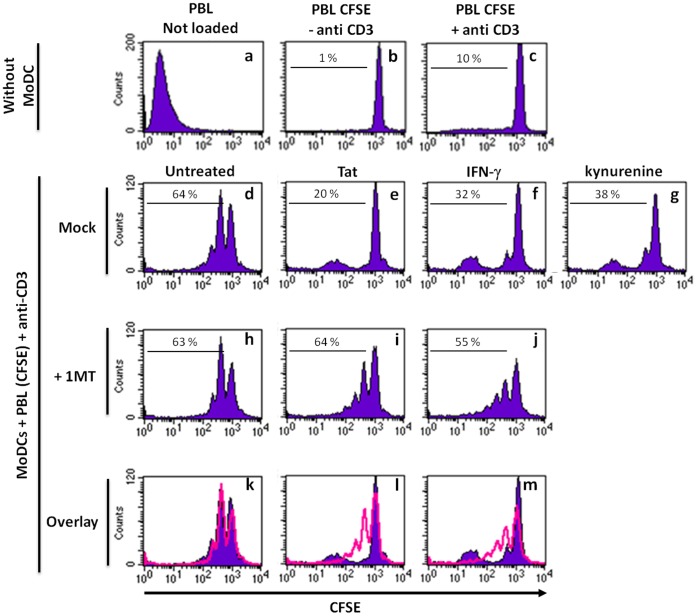
Tat treated MoDC inhibits T cell proliferation in an IDO dependent mechanism. Immature MoDCs were incubated for 48(50 nM), IFN-γ (500 ng/ml), or PBS at 37°C. After washing with PBS, MoDCs were put back into culture with or without IDO inhibitor 1MT (500 µM). After 2 hr, 2.10^5^ MoDCs were cocultured with 4.10^5^ autologous PBL (a CD14 negative fraction) labelled with 2 µM CFSE, and stimulated with anti-CD3, OKT3, Mab (10 ng/ml) or left unstimulated. Direct treatment with kynurenine (500 µM) was used as a control. After 5 days, autologous T cell proliferation was monitored by FACS analysis by following CFSE dilution analysis in the CD3 positive population. Overlay show T cell proliferation performed in the absence (purple) or in the presence (red) of 1MT. Histogram plots are representative of two independent experiments.

To assess the functional relevance of IDO induced by Tat, we analysed the capacity of Tat-stimulated MoDCs to activate lymphocyte proliferation. To this end, MoDCs pre-treated with Tat were cocultured with autologous PBL, that were loaded with CFSE, in the presence of a suboptimal amount of anti-CD3, OKT3, Mab. Control experiments showed that, in the absence of MoDCs, anti-CD3 antibodies alone induced a low T lymphocyte proliferation (10%) (Figure 5 a–c). In contrast, a more significant T cell proliferation (64%) was obtained when PBL were coculture with MoDCs in the presence of anti-CD3 antibodies (Figure 5d). Treatment of MoDCs with Tat protein prior to coculture led to a significant inhibition of lymphocyte proliferation resulting in a decrease from 64% to 20% (Figure 5e). Similarly, treatment with IFN-γ inhibited T-cell proliferation which shifted from 64% to 32% (Figure 5f). To understand the relationship between Tat, IDO expression and the effect of its metabolites on cell proliferation, we tested the effect of kynurenine on lymphocyte proliferation. Addition of kynurenine to the MoDC-PBL coculture inhibited T cell proliferation at similar levels of those observed with Tat protein or IFN-γ (from 64% to 38%) (Figure 5g). More interestingly, addition of 1-MT, a known inhibitor of the IDO pathway, abolished Tat inhibitory effect and restore optimal cell proliferation to 64% (Figure 5i and 5l). Similar results were observed with IFN-γ-positive control performed in the presence of 1-MT (55% instead of 32%) (Figure 5j, m). As control, we showed that 1-MT had no direct effect on T cell proliferation stimulated with anti-CD3 in the presence of Tat untreated MoDCs (Figure 5h, 5k). In contrast when the coculture was performed in the presence of anti-IL-10 neutralizing antibodies no effect on the restoration of T-cell proliferation was observed (Figure S4).

Altogether, our data suggest that, by acting on the cell membrane of human dendritic cells, HIV-1 Tat protein induces IDO expression and activity that is associated with an inhibition of lymphocyte proliferation.

## Discussion

In this study, we have shown that HIV-1 Tat protein induces the expression of IDO in monocyte-derived dendritic cells. Using Tat-deleted mutants, we showed that the Tat active domain is located at the N-terminal region 1–45 of the protein. Because this active domain lacks the basic region 47–57, which is essential for Tat internalization, we can deduce that Tat protein activates IDO production by acting at the cell membrane level. This conclusion is in agreement with several reports showing that Tat protein is able to bind to cell membrane and several receptors have been proposed by different groups, including αvβ3, and α5β1 [56], CD26 [57], CCR2, CCR3 [58] and CXCR4 [10], V-EGF and b-FGF [59] and L-Type calcium channel [60], [61], and low density lipoprotein receptor-related protein [62].

In addition to IDO, it has been shown that Tat protein is also implicated in the induction of the production of proinflammatory cytokines TNF-α [12], IL-6 [13], [14], IL-1β [15], IL-12 [16], and the anti-inflammatory, highly immunosuppressive IL-10 cytokine [17]–[19], [63]–[66]. All these cytokines, either because of their action to chronically stimulate the immune system, or because of their immunosuppressive action mediated by IL-10 and IDO are produced during HIV-1 infection. Furthermore, their amounts increase with AIDS disease progression [46], [49], [67]–[72].

Because Tat protein is also able to induce IFN-γ, a strong inducer of IDO, the production of IDO could be induced either directly by Tat or by an indirect pathway via Tat-induced IFN-γ. Complementary experiments showed that Tat induced IDO expression by a mechanism that can be considered IFN-γ independent for the following reasons: i) At a kinetic level, Tat induced IDO expression before the production of IFN-γ. However, this argument does not exclude an intracellular action of IFN-γ. ii) Treatment of MoDCs with Tat-conditioned medium was unable to stimulate IDO expression. iii) Coculture of MoDCs in a transwell cell system did not allow IDO expression in MoDCs not previously treated by Tat when they were cultured in the lower compartment. Moreover, we showed that direct contact between previously Tat treated and untreated MoDCs was not sufficient to induce IDO in untreated MoDCs. All these experiments indicate that Tat protein acts directly at the cell membrane of MoDCs to induce IDO expression. The fact that inhibitors of IFN-γ pathway, JakI and Ly294002, had no effect on the capacity of Tat to induce IDO expression in MoDCs adds new arguments in favour of Tat recruiting a new pathway different from that activated by IFN-γ. Similar conclusions were drawn by Boasso et al. in their study showing that HIV-1 was able to induce the production of IDO in plasmacytoid cells (pDC) following gp120-CD4 interaction. In addition, they showed that, while anti-CD4 antibodies were able to block IDO production, blocking of IFN-α/β or IFN-γ had no effect on the induction of IDO expression [43]. They used HIV-1MN, an X4 tropic virus, and HIV-1Ada, an R5 tropic virus, both rendered non replicative by modification with 2,2′-dithiodipyridine (2-AT). However, because this treatment inactivates only the post-binding steps in the HIV-1 cycle but has no effect on the binding and entry of HIV-1, we cannot exclude the possibility that IDO expression observed in the work of Boasso et al. [43] may have been mediated by internal viral proteins. This hypothesis cannot be excluded because Nef and Tat proteins, known for their capacity to stimulate IDO expression, were also found to be associated with HIV-1 viral particles [73], [74]. The implication of gp120-CD4 interaction in IDO production suggested in the work of Boasso et al. needs to be confirmed, at least by demonstrating that soluble gp120 of HIV-1 is also able to induce the same effect.

In HIV-1 persistent infection, an abnormal increase in the expression of IDO is often associated with several abnormalities in the balance of the immune system, such as suppression of T cell responses and impairment of the functions of antigen-presenting cells [75]–[77]. This aberrant increase of IDO expression has also been reported to be associated with an inefficient immune response against viral clearance, and seems to be associated with an expansion of the immunosuppressive T- regulatory cells, and a decrease in the population of antimicrobial Th17 cells [72]. Expansion of Treg cells is correlated with the increase of FOXP3 and CTLA4 markers, while the diminution of Th17 cell numbers parallels the progressive alteration of the mucosal barrier, leading to LPS translocation in the blood. Some authors associate LPS augmentation in the plasma of HIV-1 infected patients with its potential capacity to stimulate IDO expression [78]. However, at least *in vitro* in our hands, and as reported by others no IDO expression was observed in dendritic cells after treatment with LPS [79]. However, LPS has been reported, in some studies, to act in synergy with IFN-γ for the induction of IDO expression [72].

In agreement with the implication of IDO in the impairment of T-cell response, the present study shows that treatment of MoDCs with Tat leads to an alteration of their capacity to stimulate T cell proliferation. The fact that this inhibitory effect can be abolished in the presence of 1MT, an inhibitor of IDO activity, argues for the implication of Tat-induced immunosuppressive IDO, via the kynurenine pathway, in the inhibition of T cell proliferation. The data presented here are also in agreement with those reported by other groups and showing the capacity of kynurenine pathway inhibitors, including 1MT (1-methyl-tryptophan), to interfere with its effects both *in vitro* [37], [76], [80] and *in vivo* [81]–[83]. The first *in vivo* experiments were conducted in two different animal models. SCID (severe combined immunodeficient) mice, reconstituted with human PBMC, and then infected by intracranial administration of autologous HIV-1 infected macrophages were treated with 1MT, an IDO inhibitor, leading to a progressive elimination of HIV-1 macrophages from the brain [83]. In the more interesting model using infection with SIV (simian immunodeficiency virus) the results obtained were less clear. Despite the fact that blockade of CTLA4 in SIV infected macaques was associated with a loss of IDO production and viral load in lymph nodes [84], a more direct experiment based on direct administration of 1MT to SIV-infected macaque showed no inhibitory effect on the SIVmac-251 viral load [85]. However, in another study [82], it was shown that 1MT treatment had a beneficial effect by reducing the viral load in the group of SIV infected macaques selected for their unresponsiveness to antiretroviral therapy.

Overall, our study has shown that HIV-1, by its Tat protein, is able to specifically stimulate IDO expression/activity with the potential to inhibit MoDC-mediated T-cell proliferation. Consistently with our results, the presence of anti-Tat antibody [86] and Tat-specific cytotoxic T cells [87] have been correlated with better control of viremia and slower progression towards AIDS. This mechanism is probably not exclusive, and must be considered in association with other HIV-1 induced immunosuppressive mechanisms such as TGF-β, IL-10 and PD-1/PD-L1 [49], [88], [89]. Because Tat protein is known to be involved in the induction of some of these factors, as a pathogenic factor, it must be considered for the development of specific inhibitors and as an immunogen, for inclusion in the development of a potential anti HIV-1 vaccine candidate.

## Supporting Information

Figure S1 **Characterization of Monocytes differentiation into immature MoDCs.** Monocytes were differentiated into DCs by culture for 5 days with GM-CSF and IL-4. Differentiation was checked by monitoring the specific surface markers CD14, and CD1a, respectively present on monocytes and DCs. The immature status of DCs was verified by the expression of surface markers (CD83, CD80, CD86 and HLA-DR) by flow cytometry.
(TIF)

Figure S2 **Characterization of GST-Tat recombinant proteins.** (A) Homogeneity of GST-Tat 1–101 recombinant protein was analysed by 12% SDS-PAGE Electrophoresis. Column 1, shows GST-Tat 1–101 recombinant protein (10 µl at 5 µM). Column 2, correspond to equal volume of the un bound fraction (Eluent) following incubation of GST-Tat with anti-Tat/anti-GST coupled to protein A sepharose beads (pharmacia biotech). Column 3 shows the retained fraction that has been recovered with acetic acid treatment. (B) Equal amounts of GST, GST-Tat 1–101, GST-Tat 1–45 and GST-Tat 30–72 (1 µM) proteins were tested for trans-activation activity. HeLa cells line stably transfected with a plasmid encoding the β-galactosidase protein under the control of the LTR promoter of HIV-1 were incubated with 1 µM GST, GST-Tat 1–101 and GST-Tat 1–45 proteins. After 24 hr, cells were washed with PBS, fixed with PBS 0.5% glutaraldehyde and incubated with X-gal as b-galactosidase substrate (0.4 mg/ml X-gal, 5 mM potassium ferricyanide, 5 mM potassium ferrocyanide, 2 mM MgCl_2_). After 24 hr, the number of blue dyed cells, corresponding to transactivated cells were counted in optical microscopes magnifying 400x. The results are represented as numbers of blue cells per field.
(TIF)

Figure S3 **Absence of cytotoxic effect of PI3K and Jak I on MoDCs.** MoDCs were treated with of 1 µM Janus kinase inhibitor (JAK Inhibitor I), 20 µM phosphoinositide 3-kinases (LY 294002) inhibitor or diluent DMSO alone. After 24 h, (A) cell viability was determined by trypan blue dye exclusion. (B) The effect of chemical inhibitors on basal expression of IDO was also analysed. GST-Tat 1–101 (100 nM) treatment was used as a positive control for IDO expression. IDO protein was detected in MoDC extract by Western blotting experiments and β-actine was used as a loading control.
(TIF)

Figure S4 **IL-10 blockade do not restore the capacity of Tat-treated MoDCs to stimulate T cells proliferation.** Immature MoDCs were incubated with GST-Tat 1–101 (50 nM), GST (50 nM), or equal volume of PBS during 48 hr at 37°C. After washing with PBS, 2×10^5^ MoDCs were cocultured with 4×10^5^ autologous PBL, previously labelled with 2 µM CFSE, with or without anti-IL-10 (20 µg/ml). T cells proliferation was stimulated with anti-CD3 antibodies (10 ng/ml). After 5 days, autologous T cell proliferation was monitored by FACS analysis by following CFSE dilution analysis in the CD3 positive population. Overlay show T cell proliferation performed in the absence (purple) or in the presence (red) of anti-IL-10.
(TIF)
