# Correction: HIV-1 Tat Protein Induces the Production of IDO in Human Monocyte Derived-Dendritic Cells through a Direct Mechanism: Effect on T Cells Proliferation

**DOI:** 10.1371/annotation/d333f74d-496e-48e7-ac30-6dcfdbd7a25b

**Published:** 2013-11-19

**Authors:** Rémi Planès, Elmostafa Bahraoui

There are multiple errors in the Figure Legends of Figure 1 and Figure 2. Please see the corrected figure legends below:

**Figure 1. HIV-1 Tat induces IDO protein expression and activity in MoDCs.** Tat protein specifically induces IDO expression/activity in MoDCs. (A) MoDCs (2.106) were treated with increasing amounts (10, 100, and 200 nM) of full length recombinant GST-Tat 1-101 (SFII strain) or with the truncated forms GST-Tat 1-45, and GST-Tat 30-72 (100 nM). GST protein alone (100 nM) and IFN-γ (100 ng/ml) were used as negative and positive controls respectively. Eluent corresponds to the fraction of GST-Tat not retained following incubation of GST-Tat (100 nM) with anti-Tat/anti-GST coupled to protein A sepharose beads (pharmacia biotech). (B) MoDCs (2.106) were treated with 50 nM of Tat 1-86 protein (Lai strain) for 24 hr. Untreated and IFN-γ-treated (500 ng/ml) cells were used as negative and positive controls respectively. The specificity of Tat was evaluated by treating MODCs with Tat (50 nM) previously incubated with anti-Tat antibodies (3 µg/ml) for 30 min at 37°C. (C) MoDCs (2.106) were treated with LPS (1 µg/ml), IFN-γ (1µg/ml), GST-Tat 1-101 (50 nM), or kept untreated for 24 hr. For each experiment, the upper panel shows IDO protein expression by immuno-blot, and the loading control (β-actine) in the second line. Lower panels’ shows the tryptophan catabolism activity determined by Ehrlich’s spetrophotometric assay. (D) Intracellular IDO protein expression was assessed by flow cytometry in CD11c positive MoDCs after stimulation for 24 hr with GST (100 nM), GST-Tat (100 nM), LPS (1 µg/ml) IFN-γ (500 ng/ml), or untreated MoDC. The settings were made on the control isotype. Data are representative of three to four independent experiments.

**Figure 2. Tat induces the production of TNF-α, IL-6, IL-10, IL12, IFN-α1 and IFN-γ in MoDCs.** MoDCs (0.5x106) were incubated with increasing amounts of GST-Tat 1-101 protein (10, 50 and 100 nM). Untreated and GST-treated cells were used as controls. The capacity of Tat to induce IFN-γ was tested in the presence of anti-Tat antibodies. Untreated and LPS-treated MoDC were used as negative and positive controls respectively. After 24h, cell supernatants were harvested and analyzed for cytokine production by ELISA, including (A) TNF-α, (B) IL-6, (C) IFN-α1, (D) IL-10, (E) IL-12p70, and (F) IFN-γ. 

