# Supplementary figures and images for: HIV-1 Tat Protein Induces the Production of IDO in Human Monocyte Derived-Dendritic Cells through a Direct Mechanism: Effect on T Cells Proliferation
Source: PLoS One. 2013 Sep 20;8(9):e74551. doi: 10.1371/journal.pone.0074551 (PMC3779232; doi:10.1371/journal.pone.0074551)

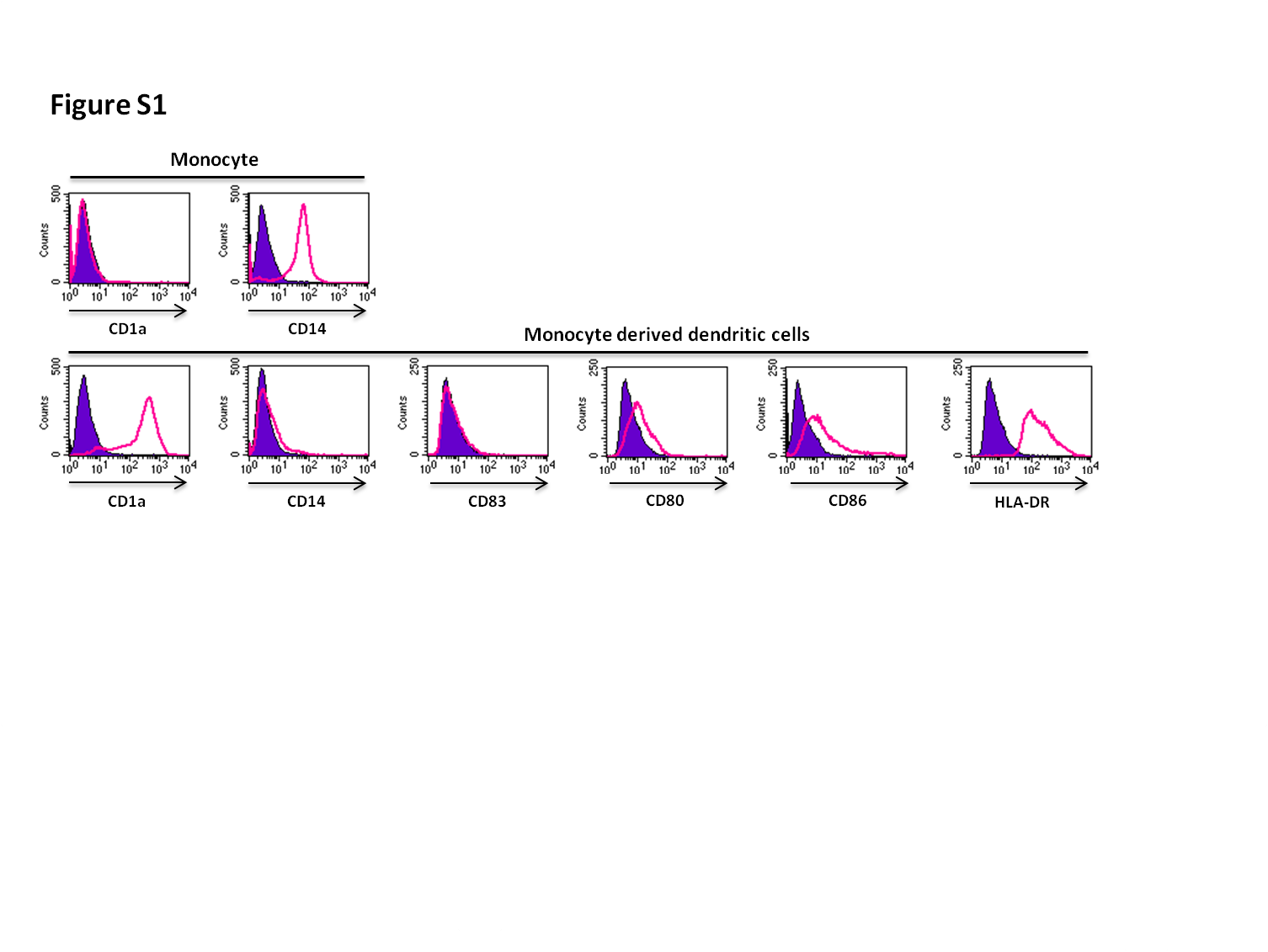

Supplement: Figure S1 — Characterization of Monocytes differentiation into immature MoDCs. Monocytes were differentiated into DCs by culture for 5 days with GM-CSF and IL-4. Differentiation was checked by monitoring the specific surface markers CD14, and CD1a, respectively present on monocytes and DCs. The immature status of DCs was verified by the expression of surface markers (CD83, CD80, CD86 and HLA-DR) by flow cytometry. (TIF) [file pone.0074551.s001.tif]

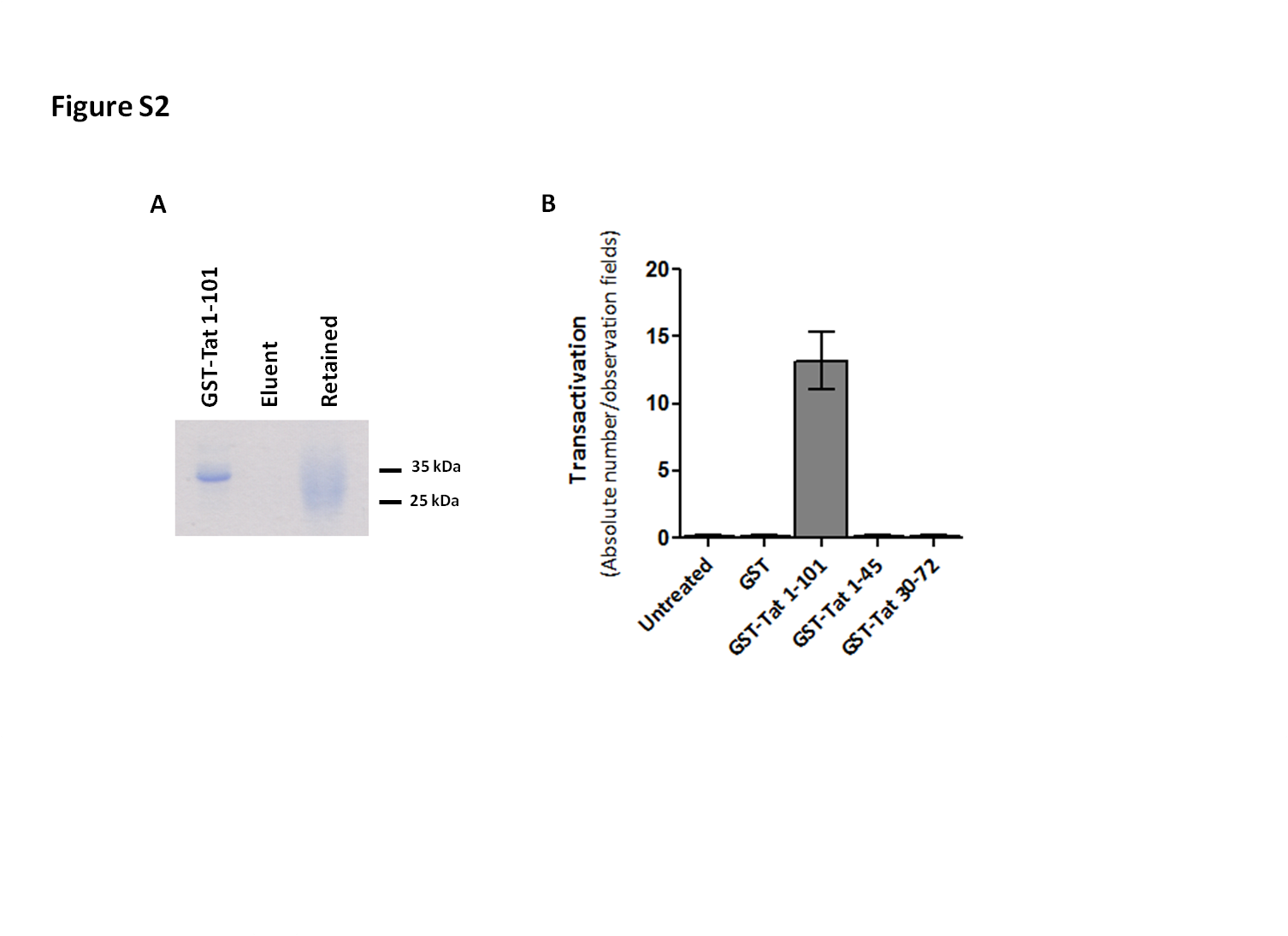

Supplement: Figure S2 — Characterization of GST-Tat recombinant proteins. (A) Homogeneity of GST-Tat 1–101 recombinant protein was analysed by 12% SDS-PAGE Electrophoresis. Column 1, shows GST-Tat 1–101 recombinant protein (10 µl at 5 µM). Column 2, correspond to equal volume of the un bound fraction (Eluent) following incubation of GST-Tat with anti-Tat/anti-GST coupled to protein A sepharose beads (pharmacia biotech). Column 3 shows the retained fraction that has been recovered with acetic acid treatment. (B) Equal amounts of GST, GST-Tat 1–101, GST-Tat 1–45 and GST-Tat 30–72 (1 µM) proteins were tested for trans-activation activity. HeLa cells line stably transfected with a plasmid encoding the β-galactosidase protein under the control of the LTR promoter of HIV-1 were incubated with 1 µM GST, GST-Tat 1–101 and GST-Tat 1–45 proteins. After 24 hr, cells were washed with PBS, fixed with PBS 0.5% glutaraldehyde and incubated with X-gal as b-galactosidase substrate (0.4 mg/ml X-gal, 5 mM potassium ferricyanide, 5 mM potassium ferrocyanide, 2 mM MgCl2). After 24 hr, the number of blue dyed cells, corresponding to transactivated cells were counted in optical microscopes magnifying 400x. The results are represented as numbers of blue cells per field. (TIF) [file pone.0074551.s002.tif]

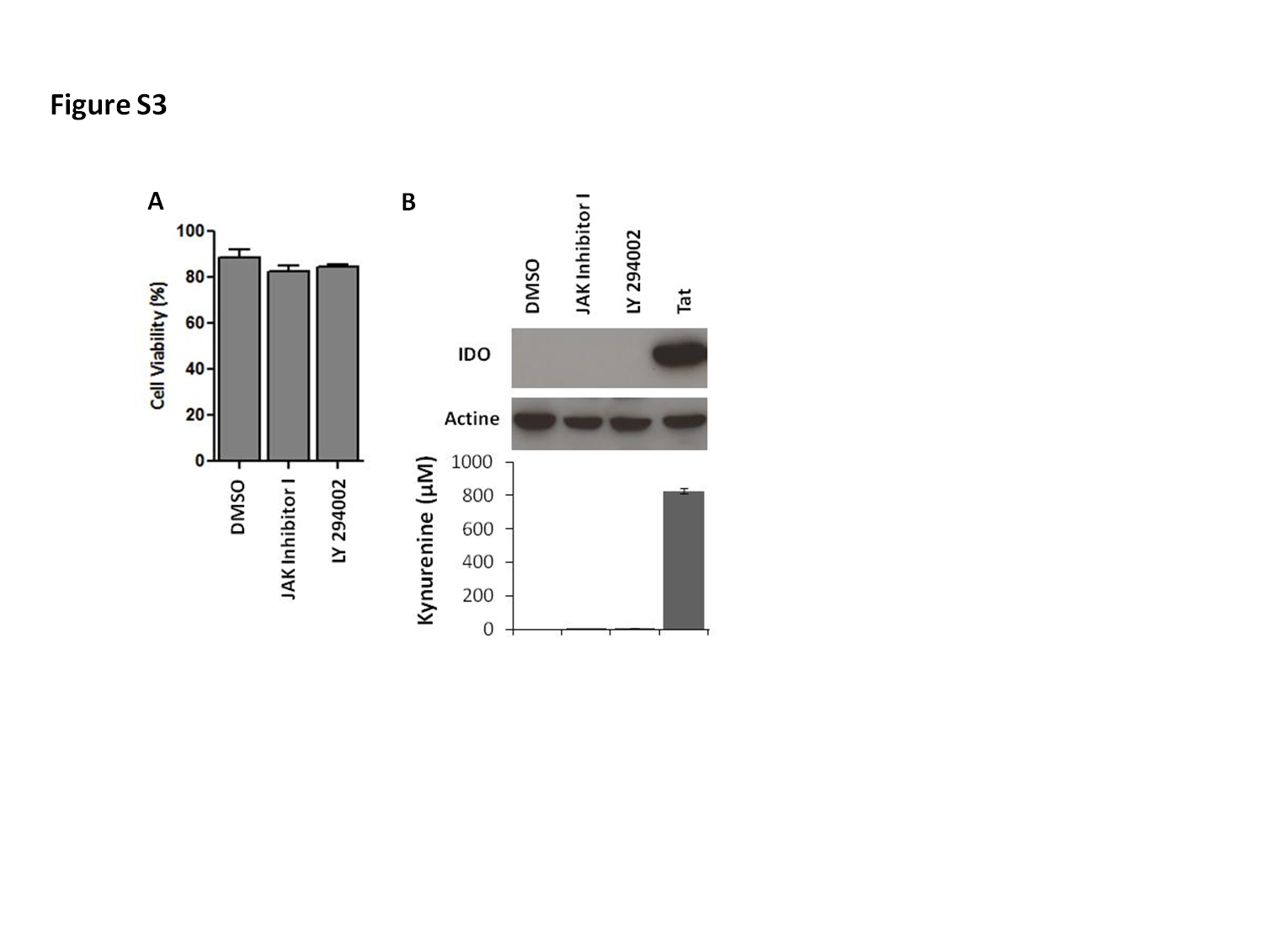

Supplement: Figure S3 — Absence of cytotoxic effect of PI3K and Jak I on MoDCs. MoDCs were treated with of 1 µM Janus kinase inhibitor (JAK Inhibitor I), 20 µM phosphoinositide 3-kinases (LY 294002) inhibitor or diluent DMSO alone. After 24 h, (A) cell viability was determined by trypan blue dye exclusion. (B) The effect of chemical inhibitors on basal expression of IDO was also analysed. GST-Tat 1–101 (100 nM) treatment was used as a positive control for IDO expression. IDO protein was detected in MoDC extract by Western blotting experiments and β-actine was used as a loading control. (TIF) [file pone.0074551.s003.tif]

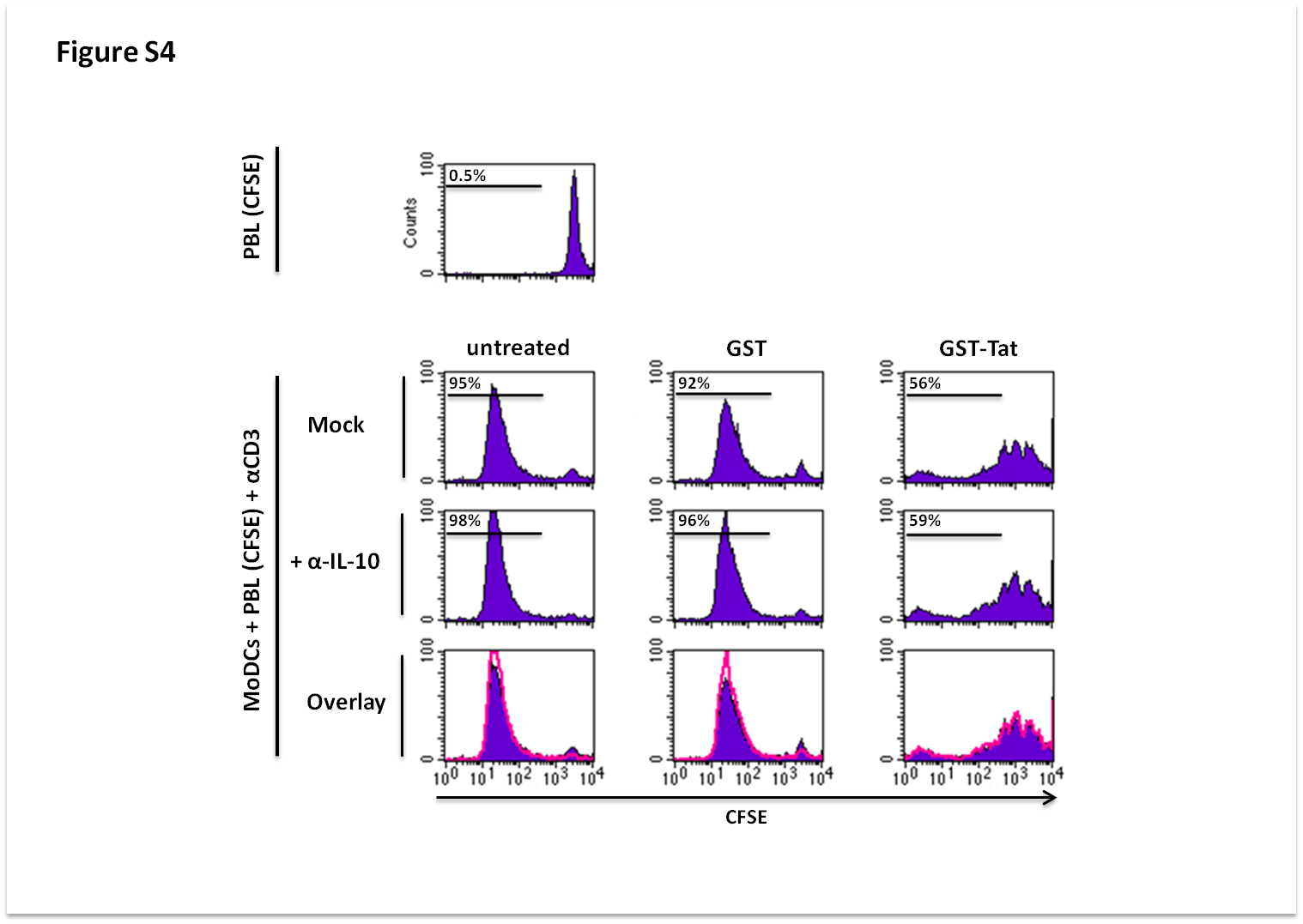

Supplement: Figure S4 — IL-10 blockade do not restore the capacity of Tat-treated MoDCs to stimulate T cells proliferation. Immature MoDCs were incubated with GST-Tat 1–101 (50 nM), GST (50 nM), or equal volume of PBS during 48 hr at 37°C. After washing with PBS, 2×10^5^ MoDCs were cocultured with 4×10^5^ autologous PBL, previously labelled with 2 µM CFSE, with or without anti-IL-10 (20 µg/ml). T cells proliferation was stimulated with anti-CD3 antibodies (10 ng/ml). After 5 days, autologous T cell proliferation was monitored by FACS analysis by following CFSE dilution analysis in the CD3 positive population. Overlay show T cell proliferation performed in the absence (purple) or in the presence (red) of anti-IL-10. (TIF) [file pone.0074551.s004.tif]
